# Breaking boundaries in ankylosing spondylitis: how innovative cell therapies reshape immunity, drive cutting-edge advances, and face future challenges

**DOI:** 10.3389/fimmu.2025.1613502

**Published:** 2025-07-11

**Authors:** Minxia Ke, Wenli Liu, Huimin Lu, Xiafei Pan, Mengyang Wu, Nianmin Qi, Zhiqiang Wang, Yuehong Wu, Feng Zhang

**Affiliations:** ^1^ Department of Biochemistry and Molecular Biology, College of Life Science and Medicine, Zhejiang Sci-Tech University, Hangzhou, Zhejiang, China; ^2^ Department of Early Research, Asia Cell & Gene Therapeutics Co., Limited, Zhejiang, China; ^3^ Department of Early Research, Horgos Stem Cell Therapy Co., Limited, Horgos, Xinjiang, China

**Keywords:** ankylosing spondylitis, mesenchymal stem cells, regenerative medicine, chimeric antigen receptor T-cell therapy, clinical progress, autoimmune inflammation

## Abstract

Ankylosing spondylitis (AS) is a chronic autoimmune inflammatory disease primarily affecting the axial skeleton, characterized by joint erosion and ankylosis. AS significantly impacts quality of life, work capacity and mental health through chronic pain, stiffness and functional decline. Its pathogenesis is multifactorial, involving genetic predispositions, immunological dysregulation and environmental triggers. Current treatments, including nonsteroidal anti-inflammatory drugs and immunosuppressive agents, offer limited symptomatic relief and fail to improve long-term prognosis due to efficacy limitations and side effects. Recent advances in cell therapy, particularly mesenchymal stem cells (MSCs) and chimeric antigen receptor (CAR) T-cell therapy, demonstrate promise in addressing these limitations by providing immunomodulatory, anti-inflammatory and regenerative benefits. This review summarizes the pathogenesis of AS, the limitations of existing treatments and the clinical progress of MSC therapy, while exploring the potential of emerging CAR-based therapies.

## 1 Introduction

Ankylosing spondylitis (AS) is a chronic autoimmune inflammatory disease predominantly affecting the axial skeleton, including the spine and sacroiliac joints, resulting in progressive joint erosion and eventual ankylosis (1). The disease progresses slowly with a long duration, and its peak onset occurs in young adults aged 20–30 years (2). The global prevalence of AS varies geographically, ranging from 0.1% to 1.4%, with a male-to-female ratio averaging 3.4:1 (3). Specifically, the prevalence rates are 0.238% in Europe, 0.167% in Asia, 0.102% in Latin America, 0.319% in North America and 0.074% in Africa. In China, a comprehensive survey across 16 regions reported an overall prevalence of 0.22%, with a male prevalence of 0.36% and female prevalence of 0.09%, yielding a male-to-female ratio of 4:1 (4). According to the latest Chinese guidelines (2022), the estimated prevalence is 0.3%, which exhibits an upward trend (5).

Clinically, AS presents with significant back pain, stiffness and functional decline, ultimately leading to spinal and pelvic fusion (6). In adolescents, AS may initially manifest as non-radiographic axial spondyloarthritis (nr-axSpA), with characteristic sacroiliac joint changes emerging later (7). AS is frequently associated with other autoimmune diseases, such as acute anterior uveitis, inflammatory bowel disease and psoriasis (8). AS exerts a lifelong detrimental effect on patients, significantly impacting their quality of life, work capacity and mental health (9). Furthermore, AS is correlated with an increased risk of premature mortality (10). In the treatment of AS, nonsteroidal anti-inflammatory drugs (NSAIDs) and immunosuppressive agents have traditionally been employed. While these therapies can effectively mitigate inflammatory responses, alleviate clinical symptoms and enhance patients’ quality of life, they are still associated with suboptimal therapeutic outcomes and a range of adverse effects (11). Moreover, current treatments fail to enhance long-term prognosis, imposing a significant burden on patients and society (12). Consequently, there is an urgent requirement for more comprehensive research into the pathogenesis of AS, alongside the expedited development of innovative therapeutic strategies.

In recent years, the emergence and advancement of innovative therapies, such as cell therapy, have offered promising new avenues for the treatment of AS. Extensive research has demonstrated that mesenchymal stem cells (MSCs) possess significant immunomodulatory and regenerative properties (13). They can mitigate inflammatory responses and facilitate tissue repair through both direct cell-to-cell interactions and the secretion of bioactive soluble factors (13). Additionally, chimeric antigen receptor (CAR) T-cell therapy has emerged as a promising therapeutic strategy for autoimmune diseases, demonstrating significant potential in early clinical trials for conditions such as rheumatoid arthritis (RA), systemic lupus erythematosus (SLE) and type 1 diabetes (14–16). This article reviews the pathogenesis of AS, existing treatment methods and their limitations, summarizes the clinical progress and mechanisms of MSC treatment for AS, and explores the potential of other cell therapies (such as CAR-based cell therapies) in the treatment of AS. Furthermore, we critically analyze the issues that need to be addressed before cell therapy can be routinely used to treat AS.

## 2 Pathogenesis of AS

The pathogenesis of AS is multifactorial, involving a complex interplay between genetic and environmental factors. Genetic factors are considered significant contributors to the development of AS, especially the human leukocyte antigen B27 (HLA-B27), which has been strongly implicated in disease susceptibility (17). The positivity rate of HLA-B27 in AS patients is over 90%, compared to only 4%-7% in the general population (17). The potential mechanisms through which HLA-B27 abnormalities contribute to the development of AS encompass: the arthritogenic peptide hypothesis, immune recognition of abnormal forms of HLA-B27, and the induction of endoplasmic reticulum stress (ERS) response due to the accumulation of misfolded HLA-B27 molecules (18).

The arthritogenic peptide hypothesis proposes that antigen-presenting cells (APCs) in AS patients present both self-antigens and microbial peptides via HLA-B27, thereby triggering a specific immune response mediated by CD8^+^ cytotoxic T cells (19). These T cells recognize and respond to the presented peptides, leading to the activation and clonal expansion of pathogenic T-cell clones that drive inflammation and tissue damage in the joints (19). A recent study has provided compelling evidence supporting the arthritogenic peptide hypothesis associated with HLA-B27 (20). This work identified CD8^+^ T cells expressing disease-related T cell receptors (TCRs) with specific TRBV9–CDR3–Jβ2.3 chains in the blood and synovial fluid of AS patients. These TRBV9 chains pair with TRAV21 chains and expand clonally within the joints. Utilizing an HLA-B27:05 yeast display peptide library, the study successfully identified microbial and self-antigen peptides capable of activating AS-associated TCRs. Structural analysis revealed that the cross-reactivity between peptide-MHC and TCRs originates from a common motif shared by self-antigens and microbial antigens, which binds specifically to the TRBV9-CDR3β TCR. These findings underscore the potential pathogenic role of both microbial and self-antigens in HLA-B27-associated diseases and highlight the arthritogenic peptide hypothesis as a key mechanism underlying the development of AS.

Abnormal forms of HLA-B27, such as homodimers, are suggested to bind to specific killer cell immunoglobulin-like receptors (KIRs) expressed on natural killer (NK) cells and CD4^+^ T cells (21–23). This interaction triggers the release of inflammatory cytokines and chemokines, thereby enhancing T cell activation and stimulating other immune cells to initiate an inflammatory response (21–23). The unfolded protein response (UPR) hypothesis suggests that the accumulation of misfolded HLA-B27 in the ER during protein biosynthesis leads to an inflammatory response (24). HLA-B27 misfolding is associated with specific polymorphisms that characterize this allele, leading to inefficient folding and peptide loading of the heavy chain (24). This misfolding can trigger ER-associated degradation (ERAD) of the heavy chains, primarily mediated by the E3 ubiquitin ligase HRD1 (SYVN1) and the ubiquitin-conjugating enzyme UBE2JL (25). Activation of the UPR has been associated with cytokine dysregulation, leading to increased production of IL-23, IFNβ, and IL-1α (26, 27). In addition to the above hypotheses, there is also evidence that HLA-B27 can disrupt the composition of the gut microbiota, leading to microbial dysbiosis, metabolic dysfunction, and loss of mucosal tolerance. This disruption can result in the release of pro-inflammatory cytokines such as IFN-γ, TNF, and IL-17, as well as the activation of regulatory T cells (Tregs) and helper T cells (Th1, Th2, and Th17 cells) (28–31). These changes contribute to chronic inflammation in the joints, skin, or gut, further complicating the pathogenesis of AS (28–31).

In addition to HLA-B27, more than 100 genes have been identified as contributing to the susceptibility of AS (32). ER aminopeptidase 1 (ERAP1) stands out as the second most significant gene associated with AS pathogenesis (33, 34). ERAP1 polymorphisms directly influence the generation of the peptide repertoire, thereby modulating the formation of pathogenic peptides that contribute to AS development (33, 34). The IL-23 receptor and the Th17/IL-23 axis are critical factors in the inflammatory cascade of AS (35). Genetic polymorphisms within these pathways have been robustly associated with disease pathogenesis, emphasizing their role in the inflammatory process. Additionally, IFNs, as key early inflammatory mediators, can induce the production of pro-inflammatory cytokines TNFα and IL-1 and activate the NF-κB signaling pathway, thereby participating in the pathogenesis of AS (36). Toll-like receptor 7 (TLR7) has also been implicated in AS susceptibility, although its role varies by sex. TLR7 acts as a protective factor in females with AS but serves as a risk factor in males, suggesting sex-specific mechanisms in disease pathogenesis (37). Additionally, the janus kinase-signal transducer and activators of transcription (JAK-STAT) pathway, a canonical signaling pathway in the inflammatory network, plays a pivotal role in AS pathogenesis (6). This pathway integrates signals from various cytokines and growth factors, driving the transcriptional response that perpetuates inflammation and tissue damage in AS (6).

Collectively, these genetic and molecular pathways underscore the complex multifactorial nature of AS, emphasizing the intricate interplay between genetic predisposition, immune signaling, and inflammatory mediators in disease development. Future research should aim to elucidate the precise mechanisms by which these genetic variants contribute to AS pathogenesis and investigate potential therapeutic targets within these pathways.

## 3 Current AS treatment options and their limitations

**Figure 1:**
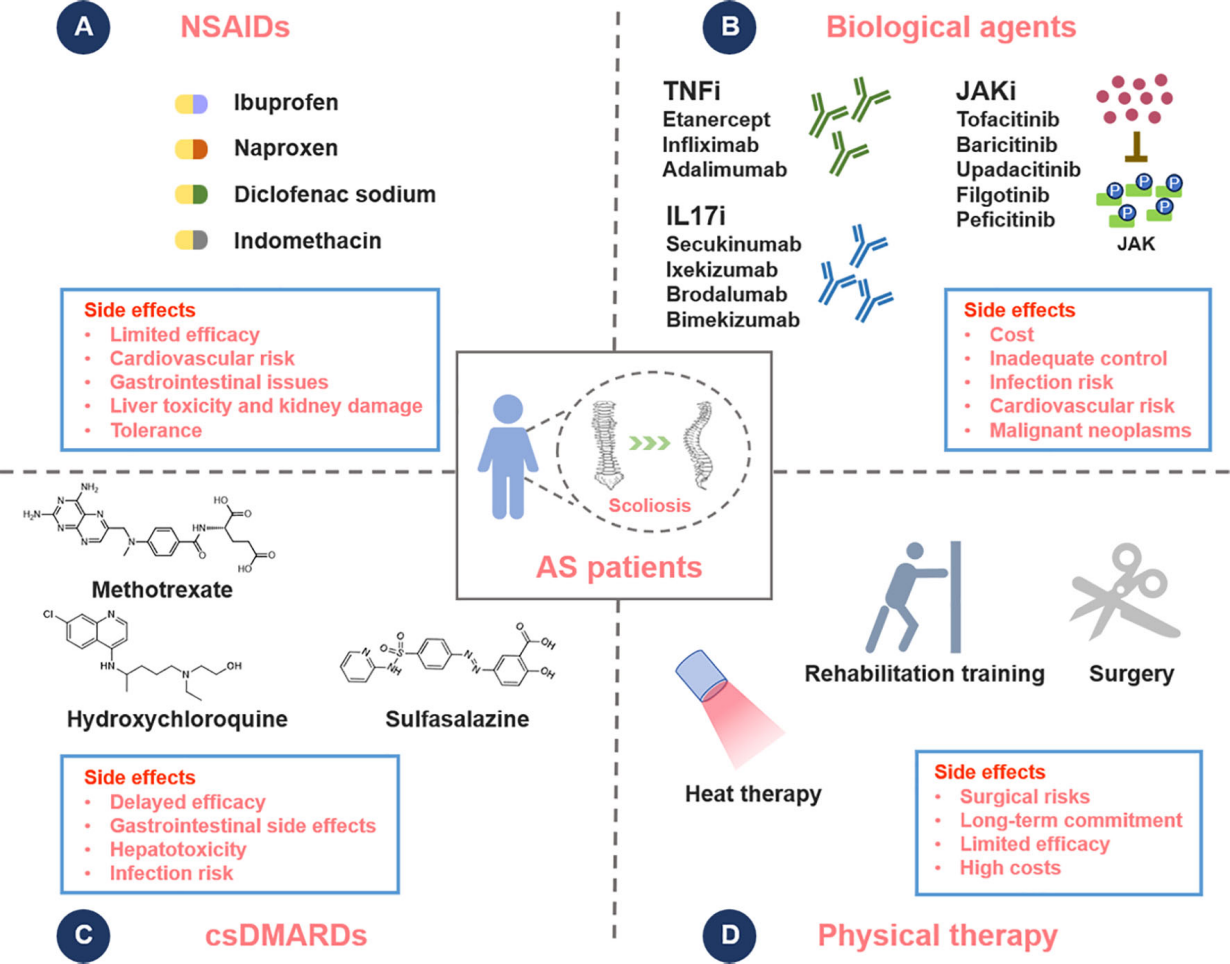
Current treatments for AS. **(A)** Nonsteroidal anti-inflammatory drugs (NSAIDs): NSAIDs are the first-line treatment for AS, providing rapid relief of back pain, morning stiffness, and joint swelling. Commonly used NSAIDs include ibuprofen, naproxen, diclofenac and indomethacin. **(B)** Biological agents: Biological agents, including TNF-α inhibitors (TNFi), interleukin inhibitors, and JAK inhibitors (JAKi), constitute a targeted and efficacious therapeutic strategy for the management of AS. These agents modulate specific inflammatory pathways, offering a more precise treatment option for patients, particularly those who exhibit an inadequate response to NSAIDs. **(C)** Conventional synthetic disease-modifying antirheumatic drugs (csDMARDs): Drugs like sulfasalazine and methotrexate are used for patients with peripheral joint involvement or those with contraindications to biologics. **(D)** Physical therapy: Physical therapy and surgical interventions are both essential components in the comprehensive management of AS. Physical therapy aims to enhance mobility and strength through personalized exercise regimens, while surgery is considered for severe cases to correct deformities or alleviate symptoms that have not responded to conservative treatments.

The treatment drugs for AS recommended jointly by the Assessment of Spondylo Arthritis International Society (ASAS), the European League Against Rheumatism (EULAR), and the Chinese Society of Rheumatology (CSR) encompass NSAIDs, biologic disease-modifying antirheumatic drugs (bDMARDs), sulfasalazine (SSZ), methotrexate (MTX), and corticosteroids (38). The efficacy of these medications varies significantly, with each class of drugs presenting distinct advantages and limitations (**Figure 1**).

### 3.1 NSAIDs

NSAIDs are the first-line treatment for AS, exerting their anti-inflammatory effects by inhibiting cyclooxygenase (COX), also known as prostaglandin endoperoxide synthase (PGHS-1 and PGHS-2) (39). These enzymes play an essential role in the biosynthesis of prostaglandins, which are key mediators of inflammation, pain, and fever. Nevertheless, despite their extensive clinical application, NSAIDs exhibit notable limitations. A recent report in Germany revealed that only 19.1% of AS patients achieved complete remission with NSAIDs (40). 30% of patients responded to NSAIDs, but many of them experienced side effects (8, 41). Long-term use of NSAIDs can induce adverse reactions in the cardiovascular, gastrointestinal, and renal systems (8, 41). Additionally, approximately one-third of patients are completely unresponsive or intolerant to NSAIDs, necessitating alternative treatment approaches (42). Consequently, bDMARDs such as TNF-α inhibitors and IL-17 inhibitors, along with Janus kinase inhibitors (JAKi), have been adopted as second-line therapies following NSAIDs failure (43).

### 3.2 Conventional synthetic DMARDs

csDMARDs are a class of drugs that can alleviate and improve symptoms in AS, including MTX, SSZ, and hydroxychloroquine (44). However, it typically takes several months to achieve therapeutic effects (44). MTX is an anti-metabolite that competitively inhibits dihydrofolate reductase, thereby interfering with DNA synthesis and modulating the expression of various cytokines (45). Patients receiving MTX should be regularly monitored for side effects through detailed questioning and frequent blood tests (46). SSZ exerts its effects by inhibiting the synthesis of prostaglandins (47). However, a recently published guideline recommends SSZ only for patients with persistent peripheral arthritis who are intolerant to or contraindicated for TNF inhibitors (48). In addition, the administration of csDMARDs at higher doses is associated with an increased risk of various adverse events, including gastrointestinal perforations, thromboembolism, and serious infections (49).

### 3.3 Targeted biological agents

#### 3.3.1 TNF inhibitors

TNF-α plays a crucial role in spondylitis and sacroiliitis, as well as in extra-articular manifestations such as uveitis (50, 51). TNF-α inhibitors (TNFi) are the most widely used and studied therapeutic agents in the treatment of AS (50, 51). Since their introduction in the early 21st century, TNFi agents have significantly improved the management of AS. Five TNF-α inhibitors are available for the treatment of AS (52, 53). Infliximab (IFX) was the first TNFi approved for treating AS. IFX is a chimeric monoclonal antibody (75% human, 25% mouse) that blocks TNF-α from activating the cellular receptor complex and is administered intravenously (IV) (54). Adalimumab (ADA), a fully humanized monoclonal antibody (IgG1), inhibits TNF-α from binding to its receptor sites and is administered subcutaneously (SC) (55). Etanercept (ETN) is a dimeric chimeric protein that combines the extracellular binding domain of human TNF receptor-2 with the Fc region of human IgG1 (56). This fusion blocks TNF from binding to cell surface receptors, inhibiting the inflammatory cascade. ETN is administered SC. Golimumab (GLM) is a fully human monoclonal antibody that specifically binds to both soluble and transmembrane TNFs, thereby inhibiting their interaction with TNF receptors (57). Administration of GLM can be performed via IV or SC routes. Lastly, Certolizumab pegol (CZP) is a PEGylated antigen-binding fragment of a recombinant human monoclonal antibody that selectively binds to and neutralizes both soluble and membrane-bound TNF-α, and is administered SC (58).

TNFi agents have demonstrated efficacy and tolerability in the treatment of AS; however, a significant number of cases have reported treatment failure. Studies have shown that approximately 35% of AS patients are primary non-responders to TNFi therapy, a condition referred to as primary clinical failure (2). Additionally, 30% of AS patients experience TNFi treatment failure within the first year of therapy (1). Notably, the rate of TNFi treatment failure is twice as high in female AS patients compared to males (59). This disparity may be attributed to differences in sex hormone balance and gene-specific expression (59). The primary cause of clinical non-response to infliximab or adalimumab is believed to be the development of antidrug antibodies (ADAs), which can affect drug bioavailability and reduce efficacy (60). The immunogenicity of biologics is unpredictable, but it can be mitigated by selecting humanized or fully human antibodies (61, 62). Beyond immunogenicity, variations in patient genetic background, disease activity, drug dosage and schedule, route of administration, concomitant medications (including immunosuppressants), and other factors all contribute to the differing sustained efficacy of each drug (60, 63).

Furthermore, TNFi treatment also brings certain side effects, limiting its applicability. AS patients with heart failure (HF) have been observed to experience worsening of their HF condition after using TNFi (64, 65). Therefore, the American College of Rheumatology (ACR) guidelines tend to recommend non-TNFi bDMARDs for treating AS patients with HF (64, 65). Infections are the most common serious adverse events associated with TNF inhibitors (66). An analysis of 71 clinical trials revealed that 40% of serious infections were attributed to the use of TNF inhibitors (67). The most common infections in IFX treatment were upper respiratory infections (24%) and skin symptoms (24%), such as itching, rash, or fungal infections (68). Other common adverse reactions included bronchitis (28%) and infusion-related symptoms (24%) (68). Moreover, the incidence of malignancies was found to be threefold higher in patients treated with IFX and ADA for rheumatoid spondylitis (69). The use of immunosuppressive drugs, including TNFi, can increase cancer risk through various pathways, with the risk varying depending on the type of cancer (69). Additionally, a significant increase in tuberculosis risk has been observed with TNFi use (70).

#### 3.3.2 IL-17/23 inhibitors

Bone marrow cells within the spine can produce IL-23 in response to mechanical stress and various other factors (71). IL-23 promotes the differentiation of Th17 cells and stimulates multiple cell types to produce IL-17 (72). Elevated levels of IL-17 and IL-23 have been observed in the peripheral blood of patients with AS compared to healthy individuals (73). IL-17A and IL-17F can amplify inflammatory responses *in vitro* when combined with TNF inflammatory regulatory factors (74). Consequently, IL-17 inhibitors, such as secukinumab, have emerged as effective second-line treatments for AS, offering significant relief of spinal pain and improved sleep quality (75, 76). However, some patients still experience treatment failure or severe side effects (74).

In a clinical study of secukinumab for AS, the most common adverse event was nasopharyngitis (11.2%), followed by mild or moderate oral candidiasis (5.3%) and serious adverse events (4.3%) (76, 77). Additionally, 6.6% of patients discontinued treatment due to adverse events (76, 77). The incidence of inflammatory bowel disease (IBD) was comparable to that observed with TNF inhibitors (76, 77). Other adverse reactions included acute uveitis, cardiovascular diseases, neutropenia, leukopenia, and staphylococcus aureus subcutaneous abscesses (76, 77). Notably, two Phase II clinical trials of IL-17 blockers for Crohn’s disease were terminated early due to worsening disease activity or a high incidence of serious adverse events (66). Therefore, AS patients with IBD or uveitis symptoms are advised to avoid IL-17 inhibitors (78).

IL-23 inhibitors initially showed promise in early studies but failed to demonstrate efficacy in Phase III clinical trials in Germany (72). Furthermore, in the treatment of AS patients with ustekinumab, an IL-23 inhibitor, it was observed that individuals at high risk for cardiovascular disease exhibited a significantly elevated risk of acute coronary syndrome and stroke (79).

#### 3.3.3 JAK inhibitors

JAK inhibitors (JAKi) interfere with the JAK-STAT signaling pathway by inhibiting one or more JAK enzymes (JAK1, JAK2, JAK3, TYK2), thereby regulating the expression of numerous inflammatory cytokines involved in autoimmune and inflammatory diseases (80). Since the approval of tofacitinib in 2012 for rheumatoid arthritis (RA), several other JAKi, including baricitinib, upadacitinib, filgotinib, and peficitinib, have been introduced into clinical practice (69). These agents have demonstrated robust efficacy in controlling disease activity, often outperforming traditional TNF inhibitors (81). However, the broad impact of JAKi on the JAK-STAT pathway, which is involved in multiple signaling cascades, raises concerns about potential off-target effects and associated safety risks.

Recent real-world clinical data and randomized trials have highlighted significant safety concerns associated with the use of Janus kinase inhibitors (JAKi). Potential serious adverse events (AEs) linked to JAKi include major adverse cardiovascular events (MACE), venous thromboembolic events (VTEs), herpes zoster, serious infections (including tuberculosis), and malignancies (82). For instance, the ORAL Surveillance trial revealed that tofacitinib was associated with a higher incidence of MACE and malignancies compared to TNFi in patients with RA (83). Additionally, tofacitinib exhibited a twofold higher risk of herpes zoster relative to bDMARDs, and this elevated risk was also observed with other JAKi (69).

These findings have prompted regulatory agencies, including the FDA and the European Medicines Agency (EMA), to issue warnings and impose restrictions on the use of JAKi, particularly in patients with cardiovascular risk factors or a history of malignancies (43). The FDA has extended boxed warnings for increased risks of MACE, VTE, infection, malignancy, and mortality to the entire class of JAKi (43). This regulatory stance underscores the critical importance of careful patient selection and individualized risk-benefit assessment when considering JAKi therapy.

Despite the availability of various treatment options, challenges persist in the management of AS. While biologics and JAK inhibitors provide substantial therapeutic benefits, they are associated with significant safety concerns, especially in patients with comorbidities such as cardiovascular disease or a history of infections. Additionally, the high costs of biologics may restrict their accessibility for certain patient populations.

### 3.4 Physical therapy

Surgical intervention may be considered for patients with AS in cases of severe spinal deformity, spinal fractures, or other significant complications when non-surgical treatments have failed. In AS, multi-level ankylosis compromises spinal stability, leading to fractures that are 3–4 times more prevalent than in the general population and predominantly affect the cervical spine or cervical-thoracic junction (84). Given the complexity, surgery is preferred over conservative treatment for better outcomes. However, it carries high risks of complications both peri-operatively and post-operatively (85). Other conventional physical therapies include cryotherapy, ultrasound therapy, electrotherapy, kinesiotherapy, and massage (86). Systematic physical activity is essential as it effectively mitigates the progression of AS. Nevertheless, physical therapy may have certain limitations, including the requirement for consistent effort and time commitment, varying effectiveness depending on individual conditions, and potentially high costs. There is an increasing emphasis on adopting a personalized and multidimensional approach to AS treatment, which integrates diverse therapeutic modalities. In light of these limitations, there is increasing interest in investigating alternative therapeutic approaches, such as cell therapy.

## 4 Mechanisms and therapeutic effects of MSCs in the treatment of AS

### 4.1 Overview of MSCs

**Figure 2:**
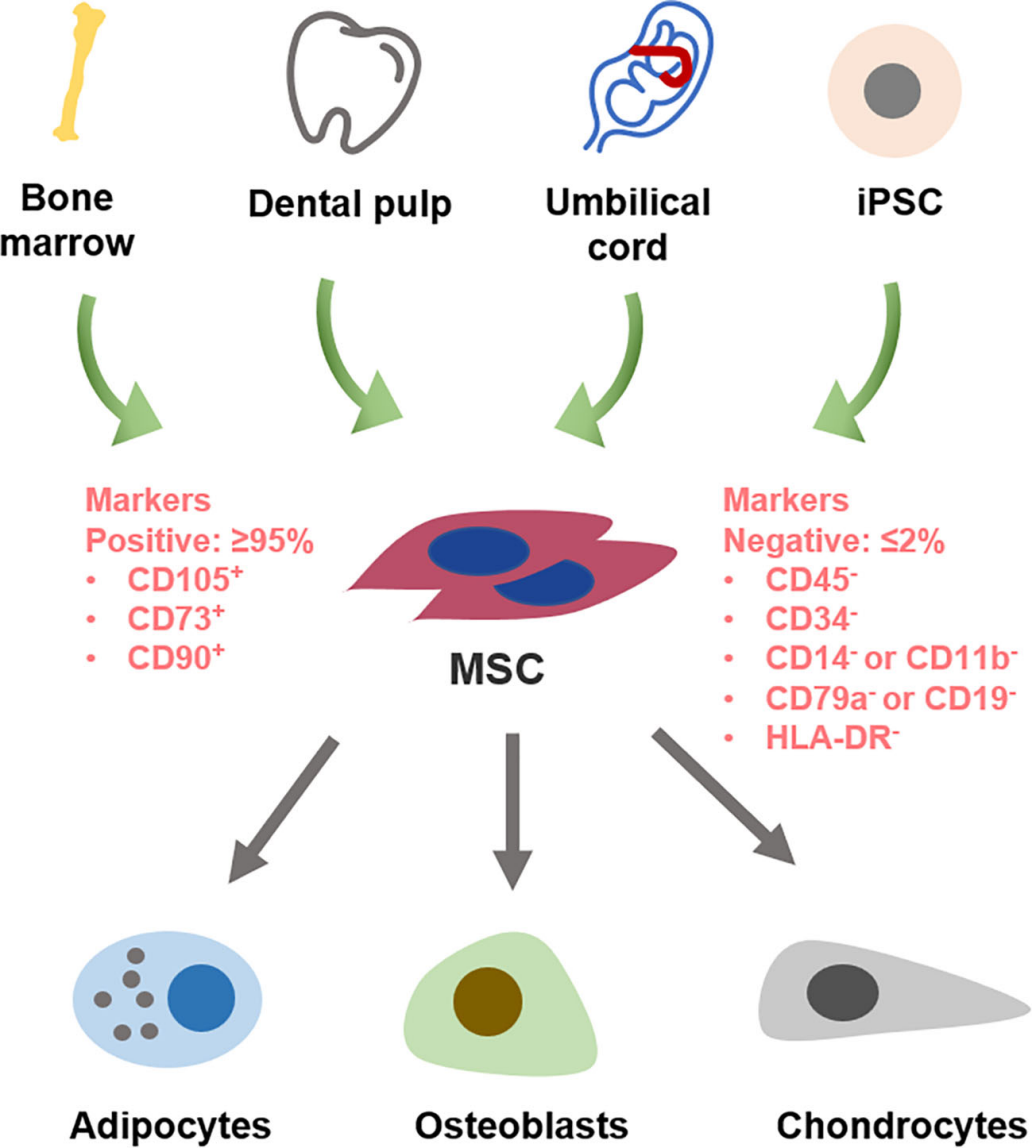
Characterization of MSCs. MSCs are derived from diverse tissue sources, including bone marrow, dental pulp, umbilical cord, and iPSCs. These cells exhibit specific surface marker expression profiles, such as CD105, CD90, and CD73, while lacking the expression of hematopoietic markers CD45, CD34, CD14 or CD11b, B cell markers CD79a or CD19, and HLA-DR. Notably, MSCs possess multipotent differentiation potential, enabling them to differentiate into various lineages, including adipocytes, osteoblasts, and chondrocytes.

MSCs are multipotent adult stem cells derived from the mesoderm during early embryonic development, characterized by their self-renewal capacity and potential for multilineage differentiation (**Figure 2**). Initially identified in bone marrow by Friedenstein et al., MSCs have since been isolated from various tissues, including umbilical cord, dental pulp, and adipose tissue (87, 88). The International Society for Cellular Therapy (ISCT) has established standardized criteria for the identification of MSCs, which include: (1) adherence to plastic *in vitro*; (2) expression of specific surface markers, such as CD105, CD90, and CD73, while lacking expression of CD45, CD34, CD14 or CD11a, CD79a or CD19, and HLA II molecules; and (3) the ability to differentiate into osteoblasts, chondrocytes, and adipocytes *in vitro* (88).

Beyond their differentiation potential, MSCs exhibit robust immunomodulatory functions, capable of modulating both innate and adaptive immune responses. They reduce the pro-inflammatory phenotype by directly or indirectly interacting with dendritic cells, macrophages, NK cells, B cells, and T cells (89). Notably, MSCs can adapt their polarization phenotypes in response to the local microenvironment, shifting between anti-inflammatory and pro-inflammatory states according to disease conditions. This adaptability makes MSCs a promising therapeutic candidate for autoimmune diseases, including AS, where the inflammatory milieu can be dynamically targeted.

### 4.2 Immunomodulatory effects and mechanisms of MSCs in the treatment of AS

MSCs are multipotent progenitor cells with the capacity to modulate immune responses and promote tissue repair through the secretion of soluble factors and direct cell-to-cell interactions. These cells exhibit potent immunosuppressive properties by secreting a variety of molecules, including indoleamine 2,3-dioxygenase (IDO), prostaglandin E2 (PGE2), hepatocyte growth factor (HGF), transforming growth factor-beta1 (TGF-β1), insulin-like growth factor-1 (IGF-1), nitric oxide (NO), heme oxygenase-1 (HO-1), cyclooxygenase-2 (COX-2), and IL-10 (90–92).

HLA-B27 is a well-established immunogenetic marker for AS, with the arthritogenic peptide hypothesis suggesting that abnormal antigen presentation to CD8^+^ T cells by HLA class I molecules triggers a specific immune response. MSCs have the ability to regulate T cell proliferation, differentiation, and activity, and can reduce the production of pro-inflammatory cytokines. MSCs can upregulate IDO expression in response to inflammatory cytokines, notably IFN-γ. IDO catalyzes the conversion of tryptophan to kynurenine, thereby inhibiting T cell proliferation through disruption of cellular protein synthesis (13, 93). Additionally, MSCs produce inducible nitric oxide synthase (iNOS), which induces macrophages to release NO, thereby suppressing T cell function (94). MSCs also inhibit the differentiation of Th17 cells, a subset of T cells implicated in the pathogenesis of AS. Huang et al. described the inhibitory effect of human umbilical cord-derived MSCs on T cells in patients with SpA (95). In co-culture with peripheral blood mononuclear cells (PBMCs), umbilical cord-derived MSCs significantly reduced the production of IL-17, showing potential for the treatment of SpA. Regulatory T cells (Tregs) are a subset of T cells with potent immunosuppressive functions, acting by suppressing effector T cells and mitigating inflammation-induced tissue damage. Both peripheral blood and synovial fluid examinations in AS patients have shown a reduced number of Tregs, which is positively correlated with lower FOXP3 expression levels (96, 97). Multiple studies have shown that MSCs induce Treg proliferation, a key mechanism by which they limit inflammation. For instance, bone marrow-derived MSCs promote the differentiation of CD4^+^ T cells into Tregs in co-culture with PBMCs, expressing high levels of CD25 and FOXP3 (98). Moreover, bone marrow-derived MSCs induce Treg proliferation through the secretion of TGF-β1 and interaction with macrophages (99). IDO is also implicated in MSC-induced Treg generation (100). MSCs can directly interact with T cells, exhibiting the most potent inhibitory effects on activated T cells through direct cell-to-cell contact (101). This interaction is further enhanced by the upregulation of intercellular adhesion molecule-1 (ICAM-1) and vascular cell adhesion molecule-1 (VCAM-1) in MSCs, which strengthens their engagement with T cells (101).

Monocytes and macrophages in AS can polarize into pro-inflammatory (M1) or anti-inflammatory (M2) phenotypes, a process closely related to active inflammation, tissue damage, and regenerative reconstruction. In late-stage AS patients, monocytes were significantly polarized into M2 macrophages, with the M2/M1 ratio positively correlated with structural lesion damage (mSASSS) and negatively correlated with inflammatory markers (ESR, CRP) and the Bath Ankylosing Spondylitis Disease Activity Index (BASDAI) (102). MSCs influence the polarization of macrophages, which may be caused by cell-to-cell contact mechanisms and soluble factors (such as IDO, PGE2, IL-10, and COX-2) (13). For example, MSCs inhibit the proliferation of M1 macrophages and activate the production of M2 macrophages through the activation of TNF-mediated COX-2 and TNF-stimulated gene 6 (TSG-6) (13). Our previous work also demonstrated that in a mouse spondylitis model, the injection of umbilical cord-derived MSCs reduced the levels of inflammatory cytokines (TNF-α and CCL-2) in the spleen and serum of mice (103).

NK cells are a critical component of the innate immune system. HLA-B27 is specifically recognized by the inhibitory receptor KIR3DL1 on NK cells, with a correlation between KIR receptor expression and AS activity (104). This suggests that NK cells play a significant role in AS pathogenesis. MSCs can regulate NK cell phenotype through cell-to-cell interactions or secretion of factors such as TGF-β1 and PGE2, inhibiting their proliferation, cytokine secretion, and cytotoxicity (105). MSCs also suppress IL-2-stimulated NK cell proliferation (106). Interestingly, MSCs secrete HLA-G5 and IFNγ, which inhibit NK cell cytotoxicity and innate immune responses while promoting Treg proliferation (90).

Dendritic cells (DCs) are key antigen-presenting cells that synthesize IL-23, a major pro-inflammatory cytokine in AS (26, 107). IL-23 induces the differentiation of lymph node T cells into pro-inflammatory Th17 cells and stimulates IL-23R^+^ lymphocytes in the sacroiliac joints to secrete IL-22, which in turn activates osteoblasts and leads to local bone formation (108, 109). MSCs inhibit the upregulation of antigen-presenting and co-stimulatory signals (CD1a, CD40, CD80, CD86, and HLA-DR) during DC differentiation and prevent the increase in CD40, CD86, and CD83 expression during DC maturation (110). Moreover, MSCs and their supernatants interfere with DC endocytosis, reducing their ability to secrete IL-12 and activate allogeneic T cells (110). Jiang et al. also proposed a similar view that MSCs can reduce the expression of CD83 on mature DCs, indicating that DCs have lost their mature characteristics (111). MSCs can also inhibit DC maturation stimulated by CSF and IL-4 through the secretion of PGE2 (111). Additionally, MSCs inhibit DC differentiation through the production of IL-10 and cell-to-cell contact (112).

### 4.3 Heterotopic ossification (HO): a potential mechanism of MSCs in the treatment of AS

HO represents a pathological condition defined by the ectopic formation of new bone tissue in soft tissues beyond the normal skeletal system, typically evidenced by the presence of osteoblasts and chondrocytes. HO is one of most significant pathological features of AS (113). In AS, HO is predominantly manifested in soft tissues such as spinal ligaments and tendons, where the appearance of chondrocytes leads to the development of new bone (113). This process commonly occurs in conjunction with the progression of inflammation and bone erosion observed in AS patients. It can lead to joint stiffness, spinal ankylosis, and spinal deformity, and may even result in the “folded person” phenomenon. Although inflammation has long been considered a trigger for HO in AS, existing AS treatments such as NSAIDs and TNFi can rapidly alleviate inflammation and pain, but they do not significantly prevent the progression of bone lesions in AS patients.

#### 4.3.1 Stages of HO in AS

The formation of bone tissue primarily happens through two distinct processes: intramembranous ossification and endochondral ossification (114). Intramembranous ossification is directly mediated by osteoblasts, which facilitate the local deposition of calcium phosphate crystals and subsequently contribute to bone formation (115). Endochondral ossification, which is initially mediated by chondrocytes and subsequently replaced by osteoblasts for the formation of bone tissue, plays a pivotal role in the progression of HO in AS (116).

HO in AS can be divided into four stages: inflammation, chondrogenesis, osteogenic activity, and pathological bone formation (117, 118). The initial inflammatory stage, mediated by both innate and adaptive immune cells, is a crucial trigger for HO in AS. Neutrophils from AS patients exhibited enhanced formation of neutrophil extracellular traps that carry bioactive IL-17A and IL-1β, which promote the differentiation of MSCs toward bone-forming cells (119). This inflammatory microenvironment sets the stage for subsequent pathological alterations. During the chondrogenesis stage, chondrocyte differentiation and cartilage formation occur, particularly in the ligaments of patients with early-stage AS (118). This cartilage formation serves as an intermediate phase before the onset of calcification. As the disease progresses, calcified cartilage is resorbed by osteoclasts, which are numerous in areas of ligament inflammation and on the surfaces of calcified cartilage. This osteoclast-mediated resorption of calcified cartilage initiates ossification, representing a pathologic process similar to acquired HO (118). In the osteogenic activity stage, osteoblasts replace the resorbed cartilage with bone tissue, leading to the formation of mature bone (120). As the disease progresses, approximately 60% to 70% of AS patients exhibit radiographic evidence of sacroiliac joint ankylosis, bridging ligament bone spurs in the axial skeleton, and enthesophytes or peripheral joint osteophytes (121). HO in AS is a complex and multifaceted pathological process, and understanding its stages and mechanisms is essential for developing targeted therapeutic strategies to manage HO in AS patients.

#### 4.3.2 The molecular mechanisms of endogenous MSCs in HO in AS

During bone formation, chondrocytes differentiate from MSCs and promote the recruitment and proliferation of MSCs. These MSCs subsequently differentiate into chondrocytes and osteoblasts, eventually forming a mature bone tissue structure (117). MSCs derived from AS patients exhibit enhanced osteogenic differentiation capacity, and MSCs migrating into cartilaginous tissues can promote pathological ossification by differentiating into osteoblasts. HLA-B27 promotes pathological ossification caused by AS-MSCs through the sXBP1/RARB/TNAP pathway (122). In addition to inducing ER stress, HLA-B27 accelerates bone formation by interacting with the activin receptor-like kinase-2 (ALK2) subunit of the BMP signaling pathway, thereby enhancing the sensitivity of the BMP-TGF signaling pathway to TGF-β and upregulating the expression of tissue nonspecific alkaline phosphatase (TNAP) (123). Mutations in TNAP haplotypes, including rs3767155 (G), rs3738099 (G), and rs1780329 (T), are primarily associated with ankylosis in AS (124). The ossification of AS-MSCs requires the synergistic action of HLA-B27 and TNAP, which may explain why not all HLA-B27-positive individuals develop ankylosis.

Furthermore, the reduction of DKK-1 in AS-MSCs mediated by inflammatory cytokines is a key factor in pathological bone formation. Compared with controls, MSCs from AS patients exhibit insufficient DKK-1 expression, mainly due to IL-17-mediated inhibition of DKK-1 and stimulation of osteoblast function (125). Additionally, the imbalance of BMP-2 and Noggin secretion may lead to abnormal osteogenic differentiation of AS-MSCs (126). Osteoprogenitor cells secrete chemokine ligand CXCL12 and stem cell factors, stimulating the proliferation of myeloid MSCs. Osteocytes secrete sclerostin and granulocyte colony-stimulating factor, regulating the differentiation of lymphocytes and myeloid cells (127). In summary, these studies reveal the intricate interplay between the immune and skeletal systems, with numerous common cytokines implicated in both.

#### 4.3.3 Therapeutic potential of transplanted MSCs for HO in AS

In the preceding section, numerous studies have demonstrated the immunomodulatory role of MSCs in the inflammatory process of AS. MSCs suppress inflammatory signals that are essential for osteogenesis, such as IL-17, thereby potentially inhibiting HO (128). Moreover, our previous preclinical animal experiments have shown the therapeutic effects of MSC transplantation on AS, with MSC treatment inhibiting HO, maintaining clear facet joint spaces, and slowing down structural lesions in the intervertebral disc, nucleus pulposus, annulus fibrosus, and cartilage (103). However, further in-depth exploration is still needed regarding the effects and mechanisms of MSC transplantation on AS, especially in terms of HO.

### 4.4 Clinical application of MSCs in the treatment of AS

**Figure 3:**
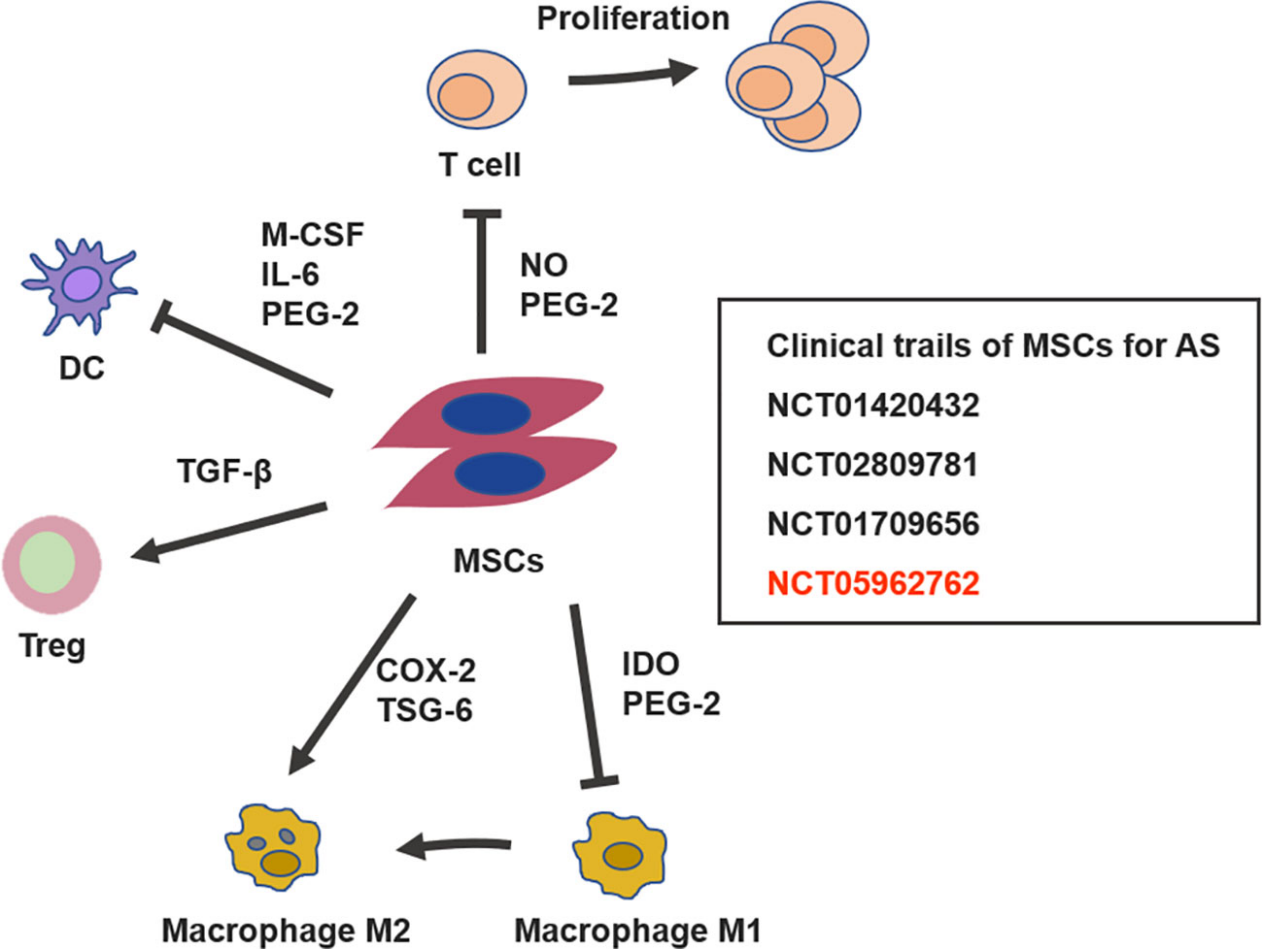
Immunomodulatory mechanisms of MSCs and current clinical trials in AS. MSCs inhibit the proliferation of T cells, promote the differentiation of regulatory T cells (Tregs), suppress dendritic cell (DC) maturation, and induce macrophages to adopt an immunosuppressive phenotype. Additionally, several clinical trials are currently underway to validate the safety and efficacy of MSCs in AS, including the ongoing trial in our research group.

In recent years, the immunomodulatory and regenerative properties of MSCs have garnered significant attention, prompting the initiation of several clinical trials to explore their therapeutic potential for AS (**Figure 3**). The earliest reported use of stem cells for AS was serendipitous: a patient with acute myeloid leukemia and AS experienced marked relief of AS symptoms and improved clinical indicators following peripheral blood stem cell transplantation (129). This patient remained symptom-free from AS for approximately 3 years post-transplantation, without the need for anti-TNF or NSAID therapy (129).

In 2013, the Wang group conducted a comprehensive study to evaluate the feasibility, safety, and efficacy of bone marrow-derived MSC therapy in 31 AS patients who were intolerant to NSAIDs (130). AS patients participating in this study received four intravenous infusions of MSCs on days 0, 7, 14, and 21, with each infusion containing 1×10^6 cells/kg. The results showed that the proportion of patients achieving ASAS20 response was 77.4% at week 4, 54.8% at week 12, and 32.3% at week 16, with a mean response duration of 7.1 weeks following the fourth infusion. The mean ASDAS-CRP score decreased from 3.6 ± 0.6 at baseline to 2.4 ± 0.5 at week 4, but increased to 3.2 ± 0.8 at week 20. MRI assessments revealed a mean total inflammatory extent (TIE) of 533,482.5 at baseline, which decreased to 480,692.3 at week 4 (p > 0.05) and further to 400,547.2 at week 20 (p < 0.05). No adverse reactions were reported. In 2017, the Li group explored the therapeutic effect of umbilical cord-derived MSCs on AS (131). In this study, umbilical cord-derived MSCs were administered via intravenous infusion to five patients with AS. The cell doses ranged from 1.2 to 3.5×10^6 cells/kg, and each patient received between 1 to 3 infusions. The study revealed that following treatment, both the Bath Ankylosing Spondylitis Disease Activity Index (BASDAI) and the Bath Ankylosing Spondylitis Functional Index (BASFI) demonstrated significant reductions. Specifically, BASDAI decreased from a baseline of 4.686 ± 0.999 to 1.880 ± 1.499 at the 3-month follow-up (P=0.014), while BASFI declined from 42.000 ± 21.213 at baseline to 10.900 ± 13.585 at the 3-month follow-up (P=0.062). However, the Bath Ankylosing Spondylitis Metrological Index (BASMI) increased nonsignificantly (P=0.676). The erythrocyte sedimentation rate decreased in 3 patients, and the C-reactive protein level was significantly reduced in 1 patient. Overall, symptoms of AS improved in all patients. No serious adverse reactions were noted; however, mild transient fever occurred in three patients within 2–6 hours post intravenous administration. More recently, we conducted a clinical study (NCT05962762) further confirmed the safety and efficacy of umbilical cord-derived MSCs for AS treatment. Other ongoing trials (NCT01420432, NCT01709656, NCT02809781) continue to evaluate the therapeutic potential of MSC infusion for AS (**Table 1**).

**Table 1:** Clinical trials for AS treatment with MSCs.

Clinical trial/Report	Study design	Cell source	Number of patients	Route of administration and doses	Follow-up time	Locations
NCT01420432	Phase I	UC-MSCs	10	UC- MSCs at a dose of 1.0 × 10^6^ MSC/kg, repeated after three months and DMARDs such as sulfasalazine, methotrexate, thalidomide for 12 months	3 months	Shandong University
NCT02809781	Phase II/III	hBM-MSCs	250	1.0 × 10^6^ MSC/kg, receive infusion per week in the first 4 weeks and every two weeks in the second 8 weeks.	12 weeks	Sun Yat-Sen Memorial Hospital of Sun Yat-Sen University
NCT01709656	Not Applicable	MSCs	120	Human-MSCs: 1.0 × 10^4-6^ cells/kg, IV on day 1 of each 14–60 day cycle, 1–6 times treatment, plus NSAIDs.	24 weeks	Sun Yat-Sen University
NCT05962762	Phase I	UC-MSC	9	Low-dose group: 1x10^6^cells/kg Medium-dose group: 3x10^6^cells/kg High-does group: 5x10^6^cells/kg	4 weeks	Asia Cell Therapeutics (Shanghai)
Report (130).	/	Allogenic MSCs	31	1x10^6^ MSCs/kg body weight in 10 ml normal saline	20 weeks	Sun Yat-sen Memorial Hospital, Sun Yat-sen University, Guangzhou, P. R. China
Report (131)	/	UMSCs	5	1.2-3.5x10^6^/kg		The Second Hospital of Shandong University

The clinical information is sourced from the ClinicalTrials.gov website (https://clinicaltrials.gov/).

MSC therapy has demonstrated significant potential in improving clinical symptoms and alleviating pain in patients with AS, with a favorable safety profile. This emerging therapeutic strategy offers a promising alternative to current treatments, such as biologics and JAK inhibitors, which are often associated with notable safety concerns and high costs. The immunomodulatory and regenerative properties of MSCs, which include the secretion of soluble factors and direct interactions with immune cells, may address the underlying pathogenesis of AS more effectively, with fewer adverse effects. However, several challenges remain to be addressed. Future research should focus on optimizing MSC sourcing, dosing, and administration routes, as well as conducting well-designed clinical trials to further validate their efficacy and safety in AS. Continued research and larger-scale clinical trials are anticipated to provide valuable insights and drive the development of this innovative treatment strategy, ultimately offering new hope for patients with AS.

## 5 CAR-based cell therapies in autoimmune diseases and their potential in AS treatment

**Figure 4:**
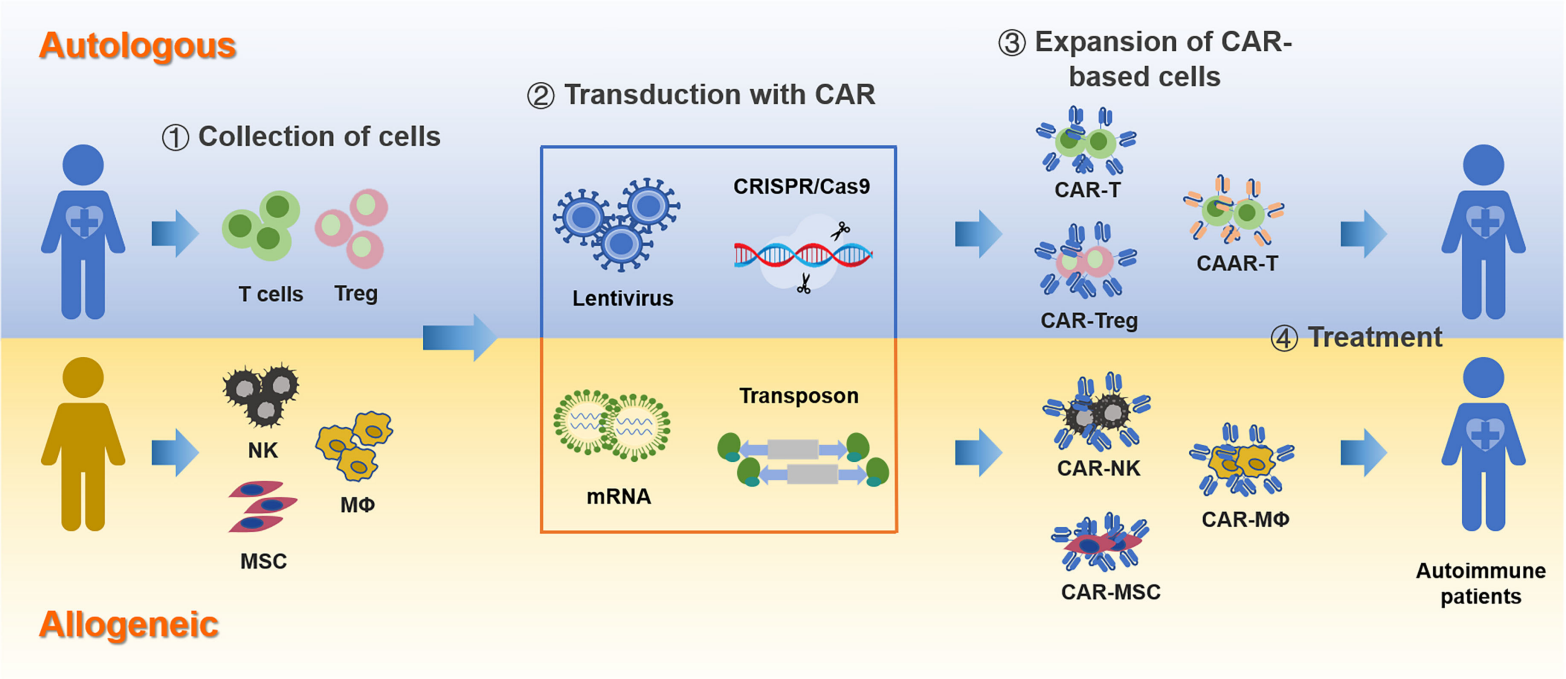
CAR-based immunotherapy for autoimmune diseases. The process of developing CAR-based therapies involves several key steps, starting from the selection of the cell source to the final deployment of engineered CAR cells.

A CAR is a chimeric antigen receptor molecule constructed through gene engineering technology, designed to confer specificity to immune effector cells, such as T lymphocytes, for a particular target antigen epitope (132). This modification enhances the ability of T cells to recognize and respond to antigen signals, thereby facilitating their activation and cytotoxic activity (132). Initially developed for cancer treatment, CAR T-cell therapy has demonstrated remarkable efficacy in managing hematologic malignancies and solid tumors. Building on these successes, CAR T-cell therapy is now being explored for its potential applications in autoimmune diseases (**Figure 4**). The rationale behind this expansion lies in the ability of CAR T cells to selectively deplete pathogenic immune cells, such as autoreactive B cells, T cells, and antigen-presenting cells (APCs), which drive the pathogenesis of autoimmune disorders. This approach aims to reset the immune system by eliminating the cells responsible for aberrant immune responses, thereby offering a novel therapeutic strategy for diseases characterized by high levels of autoantibodies or overactive lymphocytes.

### 5.1 Emerging CAR targets in autoimmune diseases

CD19 and B cell maturation antigen (BCMA) have emerged as key B-cell surface targets, demonstrating significant therapeutic potential in conditions such as systemic lupus erythematosus (SLE), idiopathic inflammatory myopathies, and systemic sclerosis (133). CD19 is expressed throughout multiple stages of B cell development, from pro-B cells to plasmablasts, but not in plasma cells (134). This widespread expression, coupled with CD19’s multifunctional role in B cell activation, maturation, and signaling, makes it an attractive target for B cell-directed therapies in autoimmune diseases such as SLE. Molecules like BCMA, CD38, and CD138 are predominantly expressed on plasma cells, with BCMA and CD38 also present on plasmablasts (134). This differential expression pattern allows therapeutic strategies to selectively target specific subsets or broader spectra of the B cell lineage, depending on the disease context and desired therapeutic effect.

Beyond B-cell targets, CAR T-cell therapies are being developed to directly target specific autoantibodies involved in autoimmune diseases. For instance, in pemphigus vulgaris, a skin disease characterized by autoantibodies against desmoglein 3 (Dsg3), anti-Dsg3 CAR T-cell therapy is currently undergoing clinical trials (135). Additionally, CAR T-cell therapies targeting cytokines are also in development, with a focus on modulating the inflammatory milieu in autoimmune diseases. Key targets include IL-23, which plays a critical role in mediating inflammatory responses (136). By targeting these cytokines, CAR T cells may potentially disrupt the pro-inflammatory signaling pathways, leading to reduced disease activity and improved clinical outcomes.

An innovative therapeutic strategy focuses on the precise elimination of pathogenic T-cell subsets that proliferate abnormally in specific autoimmune diseases. For example, targeting TRBV9^+^ T cells in AS aims to selectively eliminate pathogenic T cells while preserving normal immune cell populations (137). This strategy enhances treatment precision and reduces adverse effects on healthy cells, potentially improving the safety and efficacy of CAR T-cell therapy in autoimmune diseases. These advancements reflect the ongoing evolution of CAR T-cell therapy, moving beyond traditional cancer applications to address the complex immunopathology of autoimmune diseases. Future research is expected to identify additional targets and refine current strategies, thereby significantly broadening the therapeutic potential of CAR T-cell therapy in this field.

### 5.2 Clinical application of CAR-based cell therapies in the treatment of autoimmune diseases

Preclinical and clinical studies have demonstrated the promising therapeutic potential of CAR T-cell therapy in various autoimmune diseases, including multiple sclerosis, type 1 diabetes, inflammatory bowel disease, SLE, and pemphigus vulgaris (138). A notable case reported by the Mougiakakos group involved a woman with severe refractory SLE (SELENA score: 16) and Class III/IV lupus nephritis who received anti-CD19 CAR T-cell therapy (139). Following fludarabine lymphodepletion and CAR T-cell infusion, significant clinical improvement was observed within five weeks, characterized by normalization of dsDNA autoantibody titers and complement levels (C3 and C4). The SLE disease activity index score decreased from 16 at baseline to 0 at follow-up, and no significant adverse reactions were reported. The research team subsequently administered CAR T-cell therapy to four additional patients with refractory SLE, all of whom achieved a low lupus disease activity state (LLDAS) and successfully discontinued all SLE-specific medications (https://doi.org/10.1136/annrheumdis-2022-eular.1120). In another clinical study conducted by the Zhang group, patients with SLE and stage IV diffuse large B-cell lymphoma (DLBCL) exhibited continuous relief from disease activity following the infusion of CAR T cells targeting CD19 and BCMA (139). Follow-up examinations confirmed effective B-cell depletion, with stable disease remission lasting up to 23 months. These findings are encouraging and suggest that CAR T-cell therapy may offer a novel treatment option for patients with autoimmune diseases. However, the potential risks associated with CAR T-cell therapy, such as cytokine release syndrome (CRS) and neurotoxicity, necessitate further investigation (140). Additionally, the high cost of CAR T-cell therapy limits its widespread application.

To address these challenges, advancements in preparation techniques and diversification of cell types are being explored. Recent studies have investigated the expression of CARs in alternative cell types, such as NK cells, macrophages, regulatory T cells (Tregs), and MSCs (138). NK cells, known for their MHC-independent cytotoxicity and high safety profile, present a promising avenue for developing allogeneic therapies aimed at targeting pathogenic immune cells (141). Macrophages can phagocytose specific antigens and promote inflammatory responses, while also cross-presenting antigens to activate T cells. In contrast to the direct cytotoxic mechanisms, the activation of Tregs or MSCs through CAR-mediated antigen stimulation leverages their immunomodulatory properties to regulate immune responses. Tregs can secrete immunosuppressive molecules such as TGF-β, IL-10, and IL-35, making them suitable candidates for treating autoimmune diseases and preventing organ transplant rejection by inhibiting excessive T-cell activation (15). The Fransson group utilized CAR technology to target myelin oligodendrocyte glycoprotein (MOG) and co-express FoxP3, resulting in the generation of antigen-specific CAR Tregs (142). These MOG-CAR Tregs demonstrated the ability to inhibit effector T-cell proliferation *in vitro* and alleviate symptoms in experimental autoimmune encephalomyelitis (EAE) mouse models by reducing pro-inflammatory cytokine levels. Moreover, the MacDonald group reported that allogeneic HLA-A2 antigen-specific CAR Tregs (A2-CAR Tregs) maintained high expression levels of FoxP3, CD25, and CTLA-4 *in vitro*, effectively preventing graft-versus-host disease (GVHD) in immunodeficient mouse models (143). Recently, the Sirpilla group demonstrated the therapeutic potential of CAR-MSCs in treating GVHD (144). Specifically, E-cadherin-targeted CAR-MSCs localized to colonic cells and improved symptoms and survival rates through the upregulation of immunosuppressive genes and cytokines.

**Table 2** summarizes the main clinical progress of CAR-based cell therapies for the treatment of autoimmune diseases to date. CAR-based cell therapy has emerged as a revolutionary immunotherapy, achieving significant breakthroughs in the treatment of autoimmune diseases in recent years. These studies highlight the potential of CAR-based cell therapy to induce long-term remission and reduce disease activity in patients with severe autoimmune diseases. AS is an autoimmune disease characterized by immune system dysregulation. CAR-based cell therapy may offer new treatment opportunities for AS patients by targeting abnormal immune cells. However, the application of CAR-based cell therapy for AS is still in the research and exploration stage and has not yet reached a mature stage for clinical application.

**Table 2:** The summary of ongoing and planned clinical trials of CAR-based treatments for autoimmune diseases.

Condition	Trial registry number	Target	Cell type
SLE	NCT03030976 / NCT06150651 / NCT05988216 / NCT05859997 / NCT06333483 / NCT06056921 / NCT06420154 / NCT05859997 / NCT06222853 / NCT06347718 / NCT06294236 / NCT05765006 / NCT06361745 / NCT06417398 / NCT06152172 / NCT06121297 / NCT06297408	CD19	CAR-T cells
SLE	NCT05858684 / NCT05474885 / NCT06350110 / NCT06428188 / NCT05846347 / NCT05030779	BCMA-CD19	CAR-T cells
SLE	NCT06340490	CD19	CAR-DNT cells
SLE	NCT06373081	CD19-CD3E	CAR-T cells
SLE	NCT06153095 / NCT06462144	CD19 / CD20	CAR-T cells
SLE	NCT06249438 / NCT06316076	CD20-BCMA/ CD19	CAR-T cells / CAR-DNT cells
SLE	NCT06106906 / NCT06106893 / NCT06310811	CD19	CAR-T cells / CAR-γδT cells
SLE	NCT05869955	CC-97540 / CD-19	CAR-T cells
SS	NCT05085431	BCMA / CD19	CAR-T cells
SSc	NCT05085444	CD19 / BCMA	CAR-T cells
ANCA-associated vasculitis, AIHA (+ POEMS syndrome and amyloidosis)	NCT05263817	BCMA / CD19	CAR-T cells
MG	NCT06371040	CD19-BCMA	CAR-T cells
MG	NCT06193889 / NCT06359041	CD19	CAR-T cells
MG	NCT05828225 / NCT06419166	CD19/ CD19-BCMA	CAR-T cells
MG	NCT04146051 / NCT04561557	BCMA	CAR-T cells
MG	NCT05451212	MuSK	CAART cells
PV	NCT04422912	Dsg3 autoantibodies	CAART cells
NMOSD	NCT03605238	CD19, CD20	CAR-T cells
MG, NMOSD, CIDP, IMNM	NCT04561557	BCMAs	CAR-T cells
CD, UC, DM, AOSD	NCT05239702	CD7	CAR T cells
GVHD	NCT05993611	CD6	CAR-Tregs cells

SLE, Systemic lupus erythematosus; SS, Sjögren’s syndrome; SSc, Systemic sclerosis; MG, Myasthenia gravis; PV, Polycythemia vera; NMOSD, Neuromyelitis optica spectrum disorders; CIDP, Neuromyelitis optica spectrum disorders; IMNM, Immune-mediated necrotizing myopathy; CD, Crohn’s disease; UC, Ulcerative colitis; DM, Dermatomyositis; AOSD, Adult-onset still’s disease; GVHD, Graft-versus-host disease. The clinical information is sourced from the ClinicalTrials.gov website (https://clinicaltrials.gov/).

### 5.3 Potential of CAR-based therapy for AS treatment

In contrast to SLE, which is primarily driven by pathogenic B cells, AS is characterized by dysregulated T cell activation (145). In recent years, significant progress has been made in CD7- and CD5-targeted CAR-T cell therapy for T-cell malignancies (146, 147). However, the efficacy of these approaches in AS remains to be demonstrated. Considering the widespread distribution and critical role of T cell antigens in normal tissues, the design of CAR-T cell therapy for AS should emphasize precision to minimize potential off-target effects and preserve the integrity of the immune system. Pathogenic T cells, such as TRBV9^+^ T cells as reported, represent promising candidates for therapeutic targeting (20). Targeting these specific T cells may offer a more refined strategy for AS treatment, thereby minimizing the risk of off-target effects.

Utilizing CAR-T cells to target and eliminate pathogenic cells represents one potential therapeutic strategy for AS. Another approach involves harnessing immune regulatory cells to precisely modulate the immune microenvironment in AS. Given the limited accessibility of the disease site in AS, employing inflammation-suppressing cells such as Tregs and MSCs, with enhanced targeting capabilities, may also hold considerable promise for effectively treating AS. CAR-based therapies warrant further investigation in future studies. Leveraging CAR-based therapies to selectively eliminate the root causes of the disease while simultaneously modulating the excessive inflammatory microenvironment, without inducing significant systemic immune suppression, may offer a new generation of safe and effective therapies for curing AS.

## 6 Perspective on novel cell therapies for AS treatment

**Figure 5:**
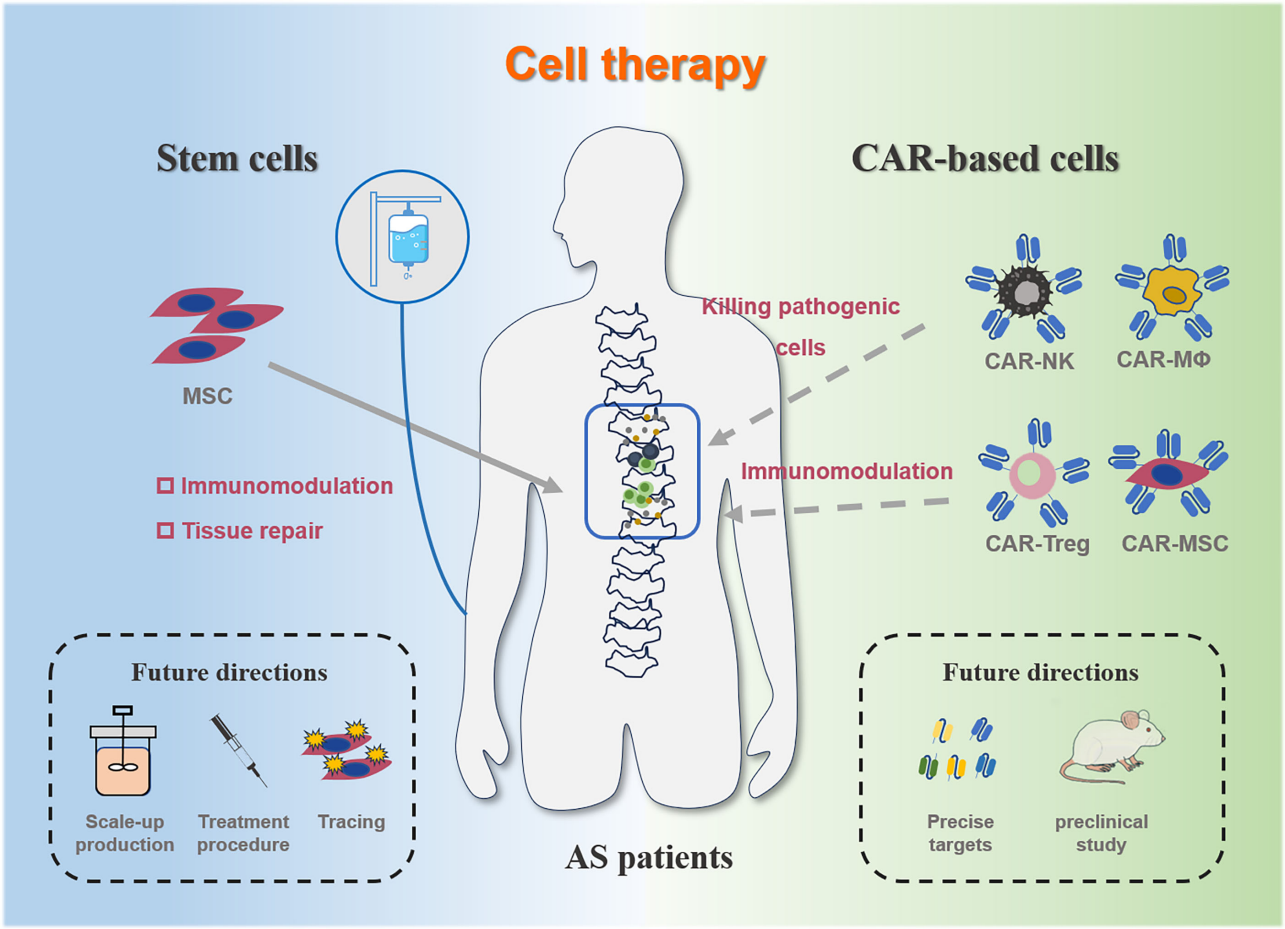
Schematic overview of current and future directions in cell therapy for AS. The left side illustrates the current schematic, mechanisms, and future directions of MSC therapy for AS, while the right side depicts the possible schematic, mechanisms, and future directions of CAR-based therapy for AS. Solid lines indicate ongoing research, and dashed lines suggest potential future directions.

Despite the availability of diverse treatment modalities for AS, many therapeutic regimens are often accompanied by challenges such as adverse effects and the development of long-term drug resistance. These challenges require us to continuously explore and develop novel treatment approaches. The emergence and development of novel cell therapies, particularly MSC therapy and CAR-based therapy, have brought promising hope to the treatment of AS (**Figure 5**).

### 6.1 Current challenges and next steps of MSCs for AS treatment

Extensive preclinical and clinical studies have demonstrated that MSCs exhibit high safety and efficacy in treating AS. These cells play a pivotal role in modulating overactivated immune cells, reducing chronic inflammation and promoting tissue repair through their anti-inflammatory and regenerative properties. However, before wide application of MSC treatment to AS, several challenges must be addressed.

#### 6.1.1 Quality and cost control of MSCs

The origin of MSCs is a significant factor. For acquisition, umbilical cord-derived MSCs (UC-MSCs) provide a more convenient and non-invasive alternative to bone marrow-derived MSCs (BM-MSCs) and adipose tissue-derived MSCs (AD-MSCs) (148). Moreover, the heterogeneity of different MSC populations must be carefully considered for clinical applications. A systematic review and network meta-analysis revealed that, autologous BM-MSCs showed the most improvement in Range of Motion (ROM) and pain relief in knee osteoarthritis patients, UC-MSC were most effective for positive Whole-Organ Magnetic Resonance Imaging Score (WORMS), and AD-MSCs were most effective for Western Ontario McMaster Universities Osteoarthritis Index (WOMAC)-positive patients (149). However, which types of MSCs have the best therapeutic outcomes for AS remain uncertain.

Recently, the U.S. Food and Drug Administration (FDA) in the United States and the National Medical Products Administration (NMPA) in China approved two MSC drugs for treating GVHD (150). However, there is a significant price difference between the Ryoncil^®^ (allogeneic BM-MSCs, Mesoblast) and Amimestrocel Injection (hUC-MSCs, Platinum Life). This discrepancy can primarily be attributed to variations in cell sources, research costs, manufacturing procedures, and market strategies. To address the cost and ensure consistent quality and efficacy, standardization of practices in culture, cryopreservation, and transportation of MSCs is essential in both preclinical and clinical settings (151). Moreover, the *in vitro* expansion of MSCs to achieve high cell yields is critical for advancing MSC therapy (151). This process involves cost challenges that must be addressed for feasible and scalable MSC treatments. Striking a balance between optimizing MSC proliferation and ensuring safety, efficacy and cost-effectiveness is essential for broader clinical application.

#### 6.1.2 Optimization of the MSC administration procedure

The ideal treatment dosage, optimization of the administration route and determination of the optimal timing for MSC intervention in AS patients should be standardized and incorporated into a standardized operating procedure (SOP) to facilitate comparisons of MSC therapy efficacy.

In a rat model of osteoarthritis, MSC transplantation via both intra-articular injection and intravenous injection was explored, with results indicating that cells administered through intra-articular injection persisted in the knee joint for up to one week, highlighting the potential for sustained local therapeutic effects (152). Current clinical trials of MSC administration for AS predominantly utilize intravenous injection, which may be limited by insufficient cell homing and retention. Exploring alternative administration routes or evaluating the potential of repeated injections represents a critical direction for advancing future research. In addition, larger-scale and higher-quality studies are needed to comprehensively evaluate the feasibility and potential value of MSC therapy for AS.

#### 6.1.3 Tracing MSC cell fate and effects *in vivo*

Although the safety of administering MSCs has been demonstrated in numerous clinical trials, the limited understanding of their dynamic biodistribution and fate within the body represents a significant challenge to the advancement of MSC therapies.

The majority of studies indicate that MSCs exhibit a relatively brief residence time in the body following intravenous administration, with most cells being sequestered in the lungs and remaining viable for 24–72 hours (153, 154). This rapid clearance is attributed to multiple factors, including apoptosis, autophagy, ferroptosis in MSCs, as well as phagocytosis by various immune cells (154–158). The fate of infused MSCs, including their interaction with the host immune system, is crucial for their therapeutic impact. MSCs are efficiently phagocytosed by innate immune cells, such as monocytes and macrophages, resulting in phenotypic and functional modifications in these cells, including the secretion of IDO and IL-10 (154, 155, 158). Innate immune cells may either remain at the initial site or migrate to other organs, thereby further regulating the adaptive immune response (154, 159). This intricate interplay of combined effects profoundly shapes the therapeutic potential of MSCs.

The development of advanced imaging and tracking technologies is crucial for elucidating the fate of MSC. In preclinical studies, precise and effective detection methods, such as magnetic resonance imaging, fluorescence labeling, optical imaging, photoacoustic imaging, ultrasound imaging and quantitative gene detection, have been widely utilized to non-invasively track transplanted stem cells (160, 161). Despite these advancements, the clinical translation of these technologies faces significant challenges. Currently, there is a lack of robust and reliable methods for tracking MSCs and their production in clinical trials. To address this challenge, the integration of multiple imaging modalities may enhance precision and provide complementary information. The development of novel imaging techniques and the identification of specific markers for MSCs are equally critical. Future progress in integrated imaging platforms, coupled with in-depth mechanistic studies, will accelerate the clinical translation of MSC-based therapies in AS.

### 6.2 The potential of precision CAR-based cell therapies for AS

CAR-based cell therapies represent a highly specific and targeted treatment modality that aligns well with the complex pathophysiology of AS. However, several critical questions still require clarification.

#### 6.2.1 Ideal targets for precision

The selection of CAR targets is of paramount importance in the development of CAR-based cell immunotherapy for AS. An ideal target antigen must exhibit high specificity and safety to minimize the risk of off-target effects leading to severe tissue damage. CAR-mediated target recognition is not limited to cell surface proteins but can also identify soluble protein ligands, post-translational modifications, and glycolipids. However, the complexity of autoimmune diseases requires careful consideration of antigen expression patterns and potential off-target effects. Unlike cancer, where CAR-T cells aim to eliminate malignant cells, CAR-based therapy for autoimmune diseases may have distinct or more complicated therapeutic mechanisms, i.e. immunomodulation. Therefore, how to selectively target pathogenic cells while sparing healthy tissues should be given more consideration. This necessitates a deep understanding of disease-specific antigen profiles and the development of CAR constructs with enhanced specificity. For instance, instead of targeting the overall T cells implicated in AS pathogenesis, targeting TRBV9^+^ T cells, a subset of T cells closed related to AS pathogenesis, provides a more precise strategy to meet the above ends.

Targeting pro-inflammatory cytokines is another strategy awaiting preclinical evaluation. A series of cytokines such as TNF-α, IL-6, and IL-17A are upregulated in AS and have a pathogenic role (162). Theoretically, using cytokine receptors as the extracellular domain of CARs could convert pro-inflammatory signals into CAR co-stimulatory signals. For instance, in tumor treatment, genetically modified CARs targeting TGF-β have been used to transmit TGF-β signals to the CD28 co-stimulatory domain, enhancing T-cell therapy (163).

#### 6.2.2 Optimal cellular candidates

The choice of CAR cells is crucial for the success of AS treatment. Tregs, known for their immunomodulatory functions, can be activated in inflammatory environments and release inhibitory cytokines such as IL-10 and TGF-β. CAR-Tregs have the potential to achieve highly effective and durable immune modulation through direct or paracrine actions, which could positively impact the disease course and prognosis of AS. Macrophages, whose phenotypes can regulate immune responses, have shown promise in treating autoimmune diseases such as type 1 diabetes when using reparative M2 macrophages (164). CAR-modified M2 macrophages may become a novel immunotherapy option for AS. Additionally, MSCs, with their potent immunomodulatory properties, could offer a new treatment paradigm for AS after CAR modification, providing higher precision and specificity. Further exploration of the roles of these cells in AS, identification of specific phenotypic markers and optimization of their regulatory functions are essential for developing new CAR therapies.

#### 6.2.3 Comprehensive preclinical validation

Before initiating multicenter clinical trials, extensive basic and preclinical research is necessary to evaluate the effects of CAR cell therapy for AS and optimize its safety and specificity. Key areas of focus include determining appropriate CAR designs and signaling mechanisms, assessing potential toxicity to normal tissues, and refining cell infusion techniques and treatment protocols. Additionally, a comprehensive evaluation of potential adverse events and long-term effects is crucial to ensure the controllability and sustainability of the treatment. By systematically conducting these preliminary studies, a solid scientific foundation can be laid for future multicenter clinical trials, thereby advancing the progress of CAR cell therapy in treating AS and making it a safer, more effective, and more sustainable treatment option.

## 7 Conclusion

The pathogenesis of AS is multifactorial, involving a complex interplay of genetic, immunological, and environmental factors. While the treatment landscape for AS has significantly evolved with the advent of advanced therapies, challenges remain in achieving long-term disease control and minimizing adverse effects. Traditional first-line treatments, such as NSAIDs and TNFis, remain the cornerstone of therapy but often fall short in addressing the heterogeneous nature of AS. The introduction of more biologic and targeted synthetic DMARDs, including IL-17A inhibitors and JAKis, has expanded therapeutic options.

Emerging cell therapies, such as MSCs and CAR-based cell therapy, offer novel approaches by targeting specific immune cells or providing regenerative benefits. These therapies hold promise in addressing the underlying pathophysiology of AS, potentially offering more durable and personalized treatment options. Nevertheless, their application in AS is still in its infancy, with ongoing clinical trials exploring their safety and efficacy.

Despite these advancements, several challenges persist. The high costs and accessibility issues associated with advanced therapies, particularly cell therapy, limit their widespread use. Furthermore, the long-term safety and efficacy of these novel approaches require further investigation through large-scale, randomized clinical trials. Future research should focus on optimizing treatment protocols, developing more precise targeting mechanisms, and exploring combination therapies to enhance efficacy and reduce side effects. Additionally, a deeper understanding of the pathogenesis of AS is crucial for the development of more effective and targeted treatments.
